# Understanding Work Addiction in Adult Children: The Effect of Addicted Parents and Work Motivation

**DOI:** 10.3390/ijerph191811279

**Published:** 2022-09-08

**Authors:** Modesta Morkevičiūtė, Auksė Endriulaitienė

**Affiliations:** Department of Psychology, Faculty of Social Sciences, Vytautas Magnus University, 44191 Kaunas, Lithuania

**Keywords:** work addiction, work motivation, children, parents, mediation analysis

## Abstract

The aim of the study was to examine the mediating role that work motivation plays in the relationship between perceived work addiction of parents and their adult child’s work addiction. The sample was comprised of 537 participants working in different Lithuanian organizations that were selected on the basis of the convenience principle. Data were collected by means of online self-administered questionnaires. To test a mediation model, a structural equation modeling was performed. It was found that perceived work addiction of both mother and father was related to higher levels of work addiction of their adult child. The results also indicated that perceived work addiction of the father was related to increased work addiction in an adult child through higher levels of extrinsic motivation as a partial mediator. The indirect effect of perceived work addiction of the mother (via extrinsic motivation) was not significant. As was expected, the indirect relationship between work addiction in parents and their adult child via intrinsic motivation was not significant. This study demonstrates that integrating both family-related and motivational variables may provide relevant insights into the nature of and mechanisms underlying work addiction and that studies in this field deserve to be further developed in future research.

## 1. Introduction

Work is an unquestionable value that brings comprehensive benefits to an individual. Work plays a key role in people’s lives providing positive aspects such as salary and financial comfort, access to the community, and new relationships [1]. Addressing things from a psychological perspective, such positive effects of work as the ability to meet important needs [2], a sense of identity and the notion of the purpose in life are worth mentioning [1]. However, due to a rising number of people over-engaged in work [3,4,5], understanding negative types of work-related behaviors has attracted a great deal of attention.

Although with some cross-cultural and cross-national variations [6], most organizations have employees who over-commit their time and energies to their working lives. Such over-commitment to work has been used in literature to describe the notion of “work addiction”. According to the authors [7], the underlying aspect of this phenomenon is a persistent psychological dysfunction. It is, therefore, not surprising that researchers think of work addiction as a negative entity. In addition to relatively mild consequences of work addiction as a lack of time for free activities [1], it has consistently been linked to such severe adverse outcomes as burnout, constant stress, work-family conflict, and lower job and life satisfaction [8]. Other negative effects of work addiction include sleep problems [9], elevated blood pressure [10], anxiety [11], depressed mood and even physical pain [12], which indicates a notable problem in living resulting from work addiction. Ultimately, it seems that it is not only an individual who suffers from work addiction, since more and more researchers (e.g., [13,14,15,16]) provide results that contribute to gainsaying the widespread belief of practitioners that work addiction may be functional from an organizational perspective.

Keeping in mind a growing scale of those displaying excessive behaviors when it comes to performing work and the negative consequences provoked by work addiction, researchers focused on analyzing its precursors. These mainly included personality traits (such as perfectionism, type A personality [8], extraversion, conscientiousness, intellect/imagination, negative affectivity, global and performance-based self-esteem [17], self-efficacy, neuroticism [18], etc.) and organizational variables (organizational climate, such as psychological climate for overwork [19,20], work characteristics, such as workload [18,21], working conditions, such as remote work [22], etc.). However, some authors (Robinson [23] probably was one of the most influential ones) noted that social influences and, more specifically, people surrounding an individual may also act as important factors inducing work addiction. Because of their authority and crucial importance in shaping a child’s attitudes and behaviors, parents were recognized as playing a key role in the development of work addiction.

Despite the interest in the overall construct of work addiction, only a few studies conducted over the past two decades were found (i.e., [24,25]) that looked into whether work addiction of parents could predict working behavior of their adult children. These scarcities are of particular concern given the studies suggesting that there is a pattern between family-of-origin issues and work habits, and that those addicted to work do not only harm themselves or negatively affect workplace outcomes but also can transmit their perceptions and compulsive work habits to their offspring [26]. Not only has relatively little research examined whether work addicted parents determine working behaviors in their children, but the few studies that have investigated this relationship provided an incomplete explanation of the issue. These studies do not completely explain the consistency and sustainability of work addiction in adult children where the addictive behavior of significant role models (i.e., parents) is no longer observed on a daily basis.

From our point of view, work addiction of a person might have deep roots in the personal aspects that are determined by parents, which shapes the persistency of work-related behaviors and attitudes towards work even if an individual is not in constant contact with the parents. Therefore, we decided to conduct the very first study where the relationship between the work addiction of parents (which was measured from the perspective of an offspring) and their adult child’s work addiction is analyzed through the mediating role of motivational forces emphasized in the self-determination theory (SDT; [27]). Theoretically, the present study has the potential to improve the understanding of work addiction by delineating underdeveloped explanatory mechanisms. From a practical perspective, our study may provide deeper insights into how to tackle the issues of work addiction at the source level and to assist in developing more nuanced prevention tools. Hence, the aim of the research was to examine the mediating role that work motivation plays in the relationship between perceived work addiction of parents and their adult child’s work addiction.

In the following sections, the theoretical framework of this study will be discussed. The research methodology and the results of statistical analysis will then be outlined. The paper concludes with the discussion of the findings obtained and research implications.

### 1.1. The Concept of Work Addiction

Conceptualizing the focal phenomenon of the present study has been a matter of great debate for decades. Despite a growing interest, no single definition or concept of it has emerged. A primary discussion develops around the terms of “workaholism” and “work addiction”. Originally, the word “workaholism” was a take on working too hard and was intended to indicate all the problems that addiction brought [28]. As a consequence, the terms “workaholism” and “work addiction” have often been used interchangeably. However, this led to a lack of conceptual and empirical clarity, and caused a construct contamination [29]. Therefore, the authors of the recent studies (e.g., [29,30]) pointed to a prospect of differentiating these two constructs. Following this idea, Morkevičiūtė and Endriulaitienė [31] performed a comprehensive quantitative literature analysis and confirmed differences in the constructs of workaholism and work addiction. Since Morkevičiūtė and Endriulaitienė [31] found the concept of workaholism to be a bit fuzzy, a decision was made to analyze work addiction in the current paper, which is defined as being overly concerned about work, being driven by an uncontrollable work motivation, and spending so much energy and effort on work that it impairs private relationships, spare-time activities and/or health [32].

When considering the phenomenon, we rely on the addiction components model that was initially proposed by Griffiths [33] and later specifically adapted to the field of excessive work by Andreassen and colleagues [32]. According to the authors, work addiction prevails where: (1) an individual is totally preoccupied with work (salience); (2) uses work to alleviate emotional stress (mood modification); (3) increases the amount of activities to achieve initial effects (tolerance); (4) experiences emotional and physical distress if is unable to work (withdrawal); (5) returns to earlier patterns of the activity after abstinence or control (relapse); (6) work comes in conflict with his/her own and others’ needs (conflict); and (7) some kind of harm or negative consequence such as a direct or indirect result of excessive working arises (problems) [32,33,34].

One of the biggest challenges about research on work addiction is to understand why people become addicted to work. Among a number of causes, addicted parents were considered to be an important factor in the development of subsequent child’s work addiction [23]. Another challenge is related to the understanding of how work addiction can be triggered by complex reasons. Trying to address this challenge, we may think of a motivational mechanism through which parents and their children are linked in terms of work addiction. As it may provide a more detailed and complete explanation of the development of work addiction, we continue to discuss this theme in the following sections.

### 1.2. Family and Work Addiction

An analysis of the literature (e.g., [35]), allows us to reject the idea that work addiction is a self-elected condition. In line with this, it can be assumed that heavy workers do not “choose” to work too much; they merely work like others in their social environment until one day they are “trapped” in their working habits. The family is where informal education begins and adult children’s behaviors at work depend largely on the attitudes and behaviors of their parents [25]. Therefore, relying on the definition given in the previous section, alongside work addiction experienced by an adult child, we decided to study the perceived work addiction of the parents.

Thus far we have found several studies conducted over the past two decades on the link between parents and children at the level of work addiction. In their study, Chamberlin and Zhang [24] examined the relationship between the work addiction of college students, the perception of the level of parental work addiction and some indicators of physical and psychological well-being. The authors found that higher levels of work addiction perceived by the children in assessing those of their parents were associated with the increased levels of children’s work addiction. More recently, when conducting a study into the sample of students and their parents, Kravina and colleagues [25] hypothesized that having at least one parent with high levels of excessive or compulsive work would be a risk factor to work addiction in adult children. The results showed that working excessively reported by adult children was significantly and positively related to working excessively reported by their fathers.

Hence, the few studies that attempted to reveal the effects of parental work addiction yielded similar results. However, more studies are needed in order to understand these still under-researched relations, especially due to a certain mismatch between the above-mentioned findings (e.g., contrary to the case of the study by Chamberlin and Zhang [24], the work-addicted mother did not cause the same addiction in a child in the study by Kravina and colleagues [25]). Therefore, we continue studies on the theme and propose the following hypotheses:

**H1a:**  *Perceived work addiction of the father is related to higher levels of work addiction of an adult child.*

**H1b:**  *Perceived work addiction of the mother is related to higher levels of work addiction of an adult child.*

### 1.3. Work Motivation in the Context of the Self-Determination Theory

Work motivation represents a set of energizing forces which initiate work-related behavior and determine its form, direction, intensity and duration [36]. Work motivation is closely related to the social context, which was proposed as a factor determining motivational outcomes [27]. Also, those who are prone to work addiction have been shown to be highly motivated, and motivation has been shown to predict work addiction [37]. Therefore, when included in the present study, the phenomenon of work motivation may be expected to provide a deeper insight into the ways in which an adult child of addicted parents becomes heavily engaged in work activities [27].

The dominant theory of work motivation—the self-determination theory—distinguishes two main types of motivation: intrinsic and extrinsic. Specifically, intrinsic motivation stems from the enjoyment and interest in the activity itself, whereas extrinsic motivation is related to performing an activity because of the need to gain some external benefits. Further, the self-determination theory distinguishes between four types of extrinsic motivation, namely, external, introjected, identified and integrated regulations. All these types differ in the level of internalization of the goals of behavior. External regulation is concerned with being motivated to obtain a desired consequence (e.g., extrinsic rewards) or to avoid an undesired one. Introjected regulation stimulates the individuals to behave in order to feel worthy, to buttress their fragile egos or to reduce such negative emotions as anxiety, guilt, shame, etc. When individuals identify themselves with the underlying value of a particular behavior, their motivational regulation is labelled as identified; in the case of identified regulation, behavior is more congruent with personal goals and identities. With integrated regulation, people have a full sense that the behavior is an integral part of who they are; actions are consistent with other values and beliefs in one’s life [27]. According to the authors [27], these regulatory types can be used individually to predict outcomes, or they can be combined to form the relative motivational index. The latter option was used in the present study.

Hence, both intrinsic and extrinsic motivations reflect different reasons for behaving, and these different reasons testify to the importance of examining types of work motivation. This provides the basis for our assumptions about the indirect relations which we discuss exhaustively in the following section.

### 1.4. The Relationship between Parents and Their Children in Terms of Work Addiction through the Mediator of Work Motivation

When analyzing indirect relations between parents’ and their children’s work addiction, it is important to discuss each pathway of these indirect relations, which might help clarify the actual mechanism we are interested in. Based on self-determination theory, as well as on the earlier studies, work-addicted parents may be expected to increase a particular type of children’s work motivation (i.e., extrinsic motivation). This is mainly because instead of letting a child feel a sense of autonomy that one’s behavior is self-chosen, work-addicted parents strongly limit their children in terms of potential behaviors and encourage them to meet strict requirements by using external stimuli [27]. In more detail, exactingness was noted as the most distinctive trait of those parents who are addicted to work [23,25]. When those addicted to work do active parenting, it is often to make sure their children are living up to their perfectionist expectations. Parents convey a message that only high achievements, together with high investments, are their desirable outcomes. Being good and doing well become the standards the children are expected to conform to and, most importantly, the love, approval and rewards of such parents are built on the condition that all these standards are reached [23]. It forms a specific motivational background towards the performance, which prevails in the working domain as a child grows up.

The following element of the mechanism of interest comprises links between work motivation and work addiction. It was proposed by some authors that the development of work addiction might result from complex motivational processes [38]. That is, the activity itself, the process or the outcome (i.e., both intrinsic and extrinsic stimuli) may form a motivational background for those addicted to work. However, in more recent studies, researchers pointed to the fact that work addiction was mainly driven by a specific type of work motivation. Although at first sight the intrinsic motivation may seem sufficient for work addiction to develop [39], and although in the beginning of developing work addiction individuals may actually have favorable intentions (e.g., to experience the joy that working activity can provide), they lose control of the behavior in the long run [35]. Despite this, the behavior continues; however, it is already maintained by external reasons (e.g., to increase self-worth, to earn recognition or social approval and to simultaneously cope with such uncomfortable emotions as anxiety, guilt or shame) [40]. This notion is in line with the findings recorded by Wojdylo and colleagues [41], who proposed that work addiction is related to strong self-control (as one of volitional modes), which operates by activating motivation via negative emotions. Hence, external factors (i.e., avoidance of negative emotions) particularly explain why those addicted to work remain so persistent in their work activities [41]. Finally, self-determination theory assumes that less desirable behavioral outcomes stem from extrinsic rather than intrinsic motivation [27]. Thus, based on all this, it can be reasonably presumed that work addiction actually has little to do with true love of one’s work; it is mainly the external stimuli that motivate a work-addicted person.

The above-listed arguments allowed us to design the measurement model of the indirect relations between parents’ work addiction and the work addiction of their adult child. When construing the model, we followed the notion that a work-addicted person is driven by strong motivational forces [35]; we continued our reasoning relying on the self-determination theory, which suggested that work motivation stemmed from the social context, that different contexts might induce different types of motivation, and that different types of motivation might induce specific behavioral outcomes [27]. In sum, in the present study we assume that work-addicted parents could hardly give rise to intrinsic motivation in their child and that work addiction most probably arises because work-addicted parents press their children to perform excessively hard and achieve a lot by employing important external stimulation. This serves as a basis for the development of work addiction, and the benefits obtained further compel a child to become addicted. Hence, we hypothesized the following:

**H2a:**  *Perceived work addiction of the father is related to increased work addiction in an adult child through higher levels of extrinsic (rather than intrinsic) work motivation.*

**H2b:**  *Perceived work addiction of the mother is related to increased work addiction in an adult child through higher levels of extrinsic (rather than intrinsic) work motivation.*

Although work motivation seems to play an important role in the relationship between parents and their children in terms of work addiction, other mechanisms could also be responsible for these relations. The mechanism explained merely by work motivation is unlikely to be thoroughly complete, since work addiction is triggered and maintained by a wide range of different factors [42]. Moreover, the possibility of a direct relationship between the parents’ levels of work addiction and the work addiction of their child cannot be denied. The direct relationship should most probably prevail even after controlling the effect of work motivation as a mediator. Therefore, we assume extrinsic motivation to act as a partial mediator in our hypothesized model.

## 2. Materials and Methods

### 2.1. Sample and Data Collection

A total of 964 employees working in different Lithuanian organizations agreed to participate in a study. However, for some reasons, a part of the sample was able to provide assessment of their parents’ working behavior basing themselves only on their memories rather than on the current case (e.g., because their parents no longer worked at the time the study was conducted, they were already dead, etc.). Different conditions under which the participants evaluated their parents resulted in measurement contamination and posed a threat to the reliability and accuracy of the data. Therefore, we eliminated a part of the sample to include only those individuals whose parents were working at the time the study was conducted. We were able to do this with the help of specific filter questions that were incorporated in the study questionnaire. Also, the participants were supposed to meet the criterion of being well acquainted with the working behavior of their parents and being able to evaluate parents in this regard; this was also controlled by using the filter questions. Hence, the final sample consisted of 537 participants. Main characteristics of the sample are shown in Table 1.

Individuals were invited to take part in the study in different ways (e.g., sending individual invitations, putting a public invitation in social media). However, the majority of the participants were invited through the secretary or the top executives of organizations. That is, first, e-mail invitations to participate in a study including information on research were sent to the organizations that were selected based on the convenience principle. Those organizations interested in participating were sent a link to the survey with a request to forward it to their employees.

The online self-administered survey included an informed consent document which, if agreed upon, automatically led to the survey instrument. The participants were informed about the aim of the study and how the data would be used. They were also assured that their responses would remain confidential, anonymous and that there were no right or wrong answers; they had to answer the questions as honestly as possible. They had the right to quit the survey anytime, as well as to revise and change their answers. It took approximately 20 min to complete the questionnaire. Participation in the study was voluntary and not rewarded.

### 2.2. Measures

To test the hypotheses, we used several previously validated instruments. Work addiction was assessed by the Bergen Work Addiction Scale (BWAS; [32]) comprising seven items, all reflecting general addiction criteria (i.e., salience, tolerance, mood modification, relapse, withdrawal, conflict, problems) experienced during the past year (e.g., “How often did you think last year about how you could free up more time for work?”). We back-forward translated the scale into Lithuanian. The scale was used to measure work addiction of a child and his/her parents as well: the participants were asked to rate their own as well as their parents’ work-related behaviors. The perceived behaviors of the mother and father were measured separately. Bearing in mind the fact that the participants might face some difficulties when assessing behaviors of their parents during the past years, we asked them to give general answers without limiting them to time boundaries (e.g., “How often does your mother think of how she could free up more time for work?”). Each item was answered on a five-point Likert scale ranging from 1 (never) to 5 (always). The scores derived from the BWAS may be used as a continuous variable or as a dichotomous variable for classifying individuals as work addicts or non-work addicts [32]. In the current study, continuous scores were used. Thus, a higher score indicated a more expressed work addiction experienced or perceived by the participant.

We used the self-determination theory-based Work Extrinsic and Intrinsic Motivation Scale (WEIMS; [36]) to measure work motivation. The Lithuanian version of this scale was prepared by Endriulaitienė and Morkevičiūtė [43]. The scale that was used in the present study consisted of 15 Likert-type items ranging from 1 (does not correspond at all) to 7 (corresponds exactly). The participants were familiarized with different reasons why work could be done, which reflected intrinsic and extrinsic motivations, e.g., “Because I derive much pleasure from learning new things”, “Because it allows me to earn money”, respectively. The participants were asked to indicate the extent to which these reasons were inherent to their case. A higher score indicated a higher level of work motivation of the participant.

Finally, the previous studies (e.g., [44]) showed that some of the socio-demographic variables might be related to the focal variables of the present study. Therefore, all participants were also asked to provide information about their gender, age, education, organizational tenure and the occupational sector.

### 2.3. Statistics

To verify the convergent validity and reliability of the constructs, the confirmatory factor analysis (CFA) using AMOS 23.0 was conducted. We employed SPSS 23.0 to generate a correlation matrix among the items, thus verifying discriminant validity. The hypotheses were also tested using SPSS 23.0 and AMOS 23.0 software. We first calculated an independent samples Student’s t-test to determine differences of the core variables among different socio-demographic groups of the participants. By doing so, we aimed at assessing which of the socio-demographic factors influenced the focal variables in our sample. This allowed us to make decisions on which of socio-demographic factors should be controlled in order to neutralize their effects when testing the hypotheses. Furthermore, partial correlations across each of the main constructs were calculated. Finally, structural equation modeling (SEM) was employed to test the mediation model. Bias-corrected bootstrap estimation (which was found to have the highest statistical power among the mediation tests [45]) was used to test the significance of indirect effects. We generated 10,000 bootstrapping samples from the original data set by random sampling. Proof of a significant indirect effect was obtained when the confidence interval of 95% did not include zero.

## 3. Results

### 3.1. Confirmatory Factor Analysis, Validity and Reliability

The measurement model of CFA had an adequate fit (χ2 (516) = 1422.02; *p* < 0.001; CFI = 0.95; TLI = 0.92; RMSEA = 0.05), considering the criteria suggested in previous studies [46,47]. All items in the model displayed acceptable standardized loadings which ranged from 0.41 to 0.96 (exceeding the acceptable value of 0.40 [48]). Average variance extracted (AVE) and composite reliability (CR) values also met their test criteria [48]: AVE values ranged from 0.50 to 0.86 (all of them exceeded or were equal to the recommended value of 0.50) and CR values ranged from 0.88 to 0.95 (they all exceeded the recommended value of 0.70). Considering all of these values, as well as the Cronbach’s alpha values, which also exceeded the recommended threshold [49], reliability and convergent validity of the instruments were confirmed (for results see Table 2).

Discriminant validity of the constructs was tested by using the heterotrait-monotrait (HTMT) ratio values [50]. The results (see Table 2) showed that all the values were below the recommended threshold of 0.90 [50]. Therefore, discriminant validity was also confirmed.

In addition, since our data were self-reported, we conducted Harman’s single-factor test [51] in order to assess the extent to which common method variance was a problem in our study. We constrained all items to load on a single-factor model. The results indicated that the fit to the one-factor model was poor (χ2 (594) = 9482.10; *p* < 0.001; CFI = 0.34; TLI = 0.30; RMSEA = 0.17).

### 3.2. Group Differences

The analysis of the independent samples *t*-test showed the main variables to be significantly related to the participants’ gender, age, education and the occupational sector. For instance, intrinsic and extrinsic motivations, work addiction of the participants themselves, as well as perceptions of work addiction of the father, were significantly related to gender. That is, female participants rated all these variables, giving them higher scores (*p* < 0.05). Furthermore, older participants reported higher levels of extrinsic motivation (*p* < 0.05). Education was related to intrinsic and extrinsic motivations, work addiction of an adult child, as well as perceptions of work addiction of the father: those with higher education reported higher levels of work addiction, intrinsic and extrinsic motivations (*p* < 0.01), whereas those without higher education rated their fathers’ work addiction by giving higher scores (*p* < 0.01). Finally, the occupational sector was related to perceptions of work addiction of the father. That is, the participants who worked in the public sector gave work addiction of their fathers higher scores than did those who worked in the private sector (*p* < 0.01). Organizational tenure was not related to the focal variables of the present study.

### 3.3. Hypothesis Testing

To establish the link between the study variables, a partial correlation was calculated. In carrying out the analysis, the participants’ gender, age, education and the occupational sector were controlled. The summary of the correlation analysis, as well as descriptive statistics, in terms of the mean and standard deviation (SD), is shown in Table 3.

The main findings in Table 3 show that perceived work addiction of both parents is significantly related to higher levels of work addiction in an adult child (*p* < 0.001), which complied with the presumptions in H1a and H1b. Before testing the mediation hypotheses, we noted a high correlation between intrinsic and extrinsic motivations. Because of high interrelations, it might have been difficult for the mediation model to estimate the relationship between each type of work motivation and work addiction independently. However, based on Gagné and Deci [27], even though intrinsic and extrinsic motivations share many qualities (and, therefore, are expected to be closely interrelated), it is important to keep these two concepts separate both theoretically and empirically. Hence, in further analyses we followed the initial idea and continued to consider these two motivational types to be separate variables.

**Figure 1:**
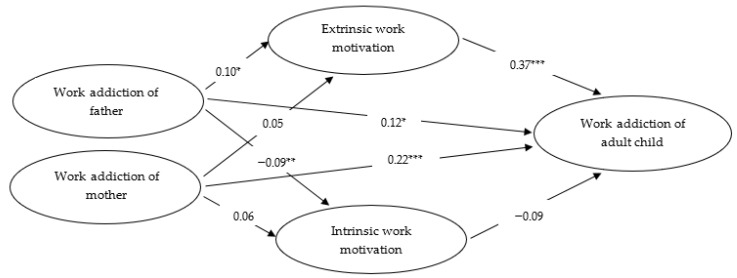
Proposed model linking perceived work addiction of parents and work addiction of an adult child (via intrinsic and extrinsic motivations). Note: The values in the model represent standardized regression weights; * *p* < 0.05, ** *p* < 0.01, *** *p* < 0.001.

In the next series of analysis, two mediation models presenting full and partial mediation were compared. Two models were generated to ascertain if the partial mediation model was truly the best-fitting one. The effects of gender, age, education and the occupational sector on all focal variables were controlled when testing both models. A full mediation model with two mediators of extrinsic motivation and intrinsic motivation where direct paths from perceived work addiction of parents to an adult child’s work addiction were constrained to zero, was initially estimated. The model showed the following fit indices: χ2 (651) = 1759.70; *p* < 0.001; CFI = 0.92; TLI = 0.90; RMSEA = 0.06; AIC = 2177.70. However, the partial mediation model revealed direct paths between the perceived work addiction of parents and an adult child’s work addiction to be significant, and based on the criteria suggested in previous studies [46,52] showed better fit indices (χ2 (608) = 1210.97; *p* < 0.001; CFI = 0.96; TLI = 0.94; RMSEA = 0.04; AIC = 1714.97). Partial mediation was thus retained for further analysis (see Figure 1).

As was expected, the indirect effect of perceived work addiction of the father on work addiction of a child (via extrinsic motivation) was significant (indirect effect = 0.016, 95% CI = [0.001, 0.041]). However, contrary to our expectations, the indirect effect of perceived work addiction of the mother (via extrinsic motivation) failed to reach statistical significance (indirect effect = 0.008, 95% CI = [−0.005, 0.029]).

As to intrinsic motivation, the latter was not a significant mediator in the relationship between parents and their children in terms of work addiction. The indirect effect of perceived work addiction of the father (via intrinsic motivation) was insignificant (indirect effect = 0.003, 95% CI = [−0.001, 0.012]). This was similar in the case with the indirect effect of perceived work addiction of the mother (indirect effect = −0.002, 95% CI = [−0.010, 0.001]).

## 4. Discussion

The literature suggests that work addiction should be considered as a construct that requires an extensive approach in order to understand it [25]; consequently, it should be analyzed in an exhaustive manner, including both the individual and situational factors. Therefore, in conducting the study we sought to shed light on the development of work addiction by analyzing both the person’s motivation and his/her perceptions about work addiction of the parents.

First, we hypothesized that perceived work addiction of the parents would relate positively to work addiction in an adult child (H1a and H1b). Our study fully confirmed H1a and H1b because it was found that perceived work addiction of both mother and father was related to higher levels of work addiction of a child. That is, the present study suggests that parents’ work addiction may be directly associated with their children’s working outcomes and that these relations do not depend on the gender of the parent. The obtained results are fully in line with the findings announced by Chamberlin and Zhang [24], and corroborate the findings of Kravina and colleagues [25] as concerns the role of the father. According to Robinson [23], parents play the central role in the formation of their children’s behaviors. The first perception of appropriate work behavior is usually gained by observing how parents behave. The model of the parents shapes the underlying patterns of attitudes, beliefs and motivations in their children’s future adulthood [25]. Because of the parents’ authority, their influence may persist even after a child grows up. Hence, it is not surprising that adult children of the parents with work addiction are more likely to exhibit problematic patterns of working behavior (which was the case in our study as well).

Second, we hypothesized extrinsic (rather than intrinsic) motivation to be fueled by the perceived work addiction of the parents and therefore to increase work addiction in an adult child (H2a and H2b). In the present study, perceived work addiction of the father was found to be related to increased work addiction of an adult child through higher levels of extrinsic motivation, which was in line with H2a. These findings supplement the propositions put forward by Kravina and colleagues [25], Robinson [23], Kim [35], Van Beek and colleagues [40], as well as the notions of the self-determination theory, based on which we assumed that substantial requests from work-addicted parents, together with the external benefits provided for fulfilling these requests, do not only increase a risk for work addiction of an adult child but also generate a real possibility for work addiction to result from extrinsic motivation. Furthermore, as we expected, the indirect relationship between work addiction in parents and their adult child (via intrinsic motivation) was not significant. Finally, even after controlling work motivation as a mediator, direct relationships between parents and their child in terms of work addiction remained significant. All of these findings confirm our assumptions about the importance of analyzing different types of work motivation when studying the development of work addiction, highlight the role of extrinsic motivation as a variable induced by the work-addicted father, comply with our considerations about partial mediation and, at the same time, provide a direction for future studies by highlighting the idea of extending the current study with a more complex viewpoint of indirect relations.

However, it should also be noted that, contrary to our expectations, the indirect effect of perceived work addiction of the mother (via extrinsic motivation) was not significant, which prevented us from confirming H2b and suggested that some other rather than motivational models might be more appropriate in explaining the influence of a work-addicted mother on an adult child’s addiction. We may think of several potential reasons for this unexpected finding. First, gender-related parenting strategies may act as an important psychological factor, since mothers and fathers might have had different parenting strategies. For instance, work-addicted mothers were possibly more flexible and warmer in their treatment of children and laid down more reasonable requirements for them, whereas fathers possibly imposed more stringent requirements on their children, making every effort to encourage them to comply with those requirements. This could be a reason for different outcomes in terms of a child’s motivation and subsequent work addiction. Second, it is also possible that the fathers were perceived as more authoritative figures as compared to mothers, which resulted in a child’s greater desire to live up to the fathers’ expectations.

Hence, the results obtained lead us to the conclusion that fathers exert a greater effect on their children in the motivational mechanism of work addiction. It means that future studies investigating the developmental mechanisms of work addiction should treat fathers’ and mothers’ work addiction separately, since fathers and mothers may each exert unique influences.

### 4.1. Theoretical Implications

To the best of our knowledge, this was the first study that integrated both the work addiction of parents and the adult child’s work motivation into the same research model and proposed, as well as tested, the potential mediation effects of work motivation on the links between the levels of work addiction of both parent and child. Hence, first and foremost, our study expands the knowledge of the field and lends highly necessary empirical support to the claims of a still under-researched link between parents and their children in terms of work addiction.

Furthermore, the results of our study are relevant to the motivation-based view, as they support the notions presented in the self-determination theory. The theory suggests that demanding and controlling social contexts lead to extrinsic (rather than intrinsic) motivation, and that extrinsic motivation is more often related to less desirable behavioral outcomes [27]. In the present study we confirmed this by showing that a work-addicted father induced merely extrinsic motivation, which in turn determined the work addiction of an adult child. Hence, we see the self-determination theory as a potential valuable framework for future investigations examining the development of work addiction in social contexts.

### 4.2. Practical Contribution

When working with clients on the issues related to work addiction, counselors may primarily help them identify their individual reinforcement history. For example, specific messages received from the parents through the lifespan should be explored. It might be necessary to confront adult children of controlling and demanding work-addicted parents and to develop new positive cues and prompts for the appropriate working behavior. In addition, counselors could teach their clients how to relax, be more flexible and spontaneous and assist them in developing an inner voice, finding out their own wants and needs.

However, interventions should target not only addicted children but also parents. Family therapy could be very useful here, so that family members (i.e., parents) become aware of how they might reinforce work addiction in a child or an adult child. Counselors should instruct parents to show unconditional regard for their children as opposed to measuring the child’s worth by what the child achieves [24].

### 4.3. Limitations and Directions for Future Research

Results from our study should be considered in light of several limitations. The first limitation is related to the existing contradictions in the conceptualization and interpretation of the focal phenomenon of our study (i.e., work addiction). Although it is generally considered as a genuine addiction [7,53], in the Diagnostic and Statistical Manual of Mental Disorders [54] it has not been formally defined as such yet. Probably one of the most significant reasons for this is the lack of clarity among different forms of excessive work [31]. Hence, future studies should, above all, make every effort to solve the conceptual issues of work addiction.

As to the study design of the present research, it should be noted that it was based on cross-sectional data. It is generally assumed that, when using this kind of design, nothing can be firmly concluded regarding the directionality between study variables. The remedy for this concern most often suggested was using a longitudinal design that introduces the element of time. However, according to the authors of the recent studies [55], the longitudinal design fails to offer many advantages over the cross-sectional design; longitudinal studies might also lead to erroneous inferences. In fact, if properly utilized, the cross-sectional design is able to provide evidence for all the main elements (or at least for some very important ones) of a causal case (for more information see [55]). Therefore, we suggest to rely on a more flexible viewpoint in evaluating particular (i.e., cross-sectional) research designs and to be open to the possibility that studies using a cross-sectional design can have a significant value. We would also like to draw attention of the researchers who are likely to continue the research line presented in this paper using longitudinal designs. The authors [55] noted that the biggest issue is that most studies that utilize longitudinal designs fail to choose time points so that causes are assessed before the effect. Rather, arbitrary time points are chosen after the underlying causal process has ended and the system has achieved a steady state (i.e., the timeframe chosen does not match the timeframe of the phenomena in question) [55]. Hence, the most important conditions that are necessary to meet are to nail down the temporal precedence and to carefully choose the lags between the occurrence of independent, dependent variables and the mediator, which can be rather challenging, especially in estimating the effects of work motivation on work addiction.

Furthermore, the exclusive use of self-report tools and the absence of other informants (e.g., parents) do not address the issue of the effect of shared method variance. Therefore, as indicated in the above sections, Harman’s single-factor test was conducted in our study. The results showed a poor fit of a single-factor model, which means that common method variance was not a major problem in our study. However, even with such results, we still cannot completely exclude the possibility that common method variance may have influenced our results [51]. Hence, future research would benefit from using a multiple source method.

Another limitation is the convenience principle that was used for selecting the sample. Although quite a large number of participants can minimize this drawback, we still recommend that researchers use a random sampling method. Moreover, as our data refer to the Lithuanian sample, generalization of the findings for other countries is difficult to make. To elucidate to what extent the results obtained can be applied to other countries, future studies should analyze samples from different populations. Another threat to the generalization of the findings stems from a rather low mean age of the sample, which may have reduced variance in data. This should be taken into account when interpreting the results of the present study and making decisions about the generalization of our findings to the new settings.

Furthermore, although we controlled the aspect of being well acquainted with the working behavior of the parents and included only those participants who were able to evaluate parents in this regard (which was primarily aimed at assisting us in achieving greater accuracy of data), as we did not collect more information, we could not control the important aspects that might determine the strength of parental influences (e.g., whether a child is still living with the parents, whether a child was living with the parents during childhood, etc.). Therefore, the links obtained might be assumed as accidental to some extent. On the other hand, the fact that a child is well acquainted with the working behavior of the parents already indicates close familial relations in general and provides support for viewing the links obtained as non-accidental and actually representing significant influences. However, in order to eliminate any doubts, our suggestion for future research within this field is to increase the scope of potentially relevant information and to collect additional data.

Lastly, the results of the present study may have been determined by the specific external circumstances to some extent. This is because the current study was carried out under difficult conditions (i.e., during the COVID-19 pandemic). As a consequence of the COVID-19 pandemic, organizations and employees had to quickly adapt to working from home [1]. Such working arrangements have been noted to be related to blurring of the boundaries between work and family life and longer working hours, which may have caused an increase in the work addiction situation [56]. The pandemic also conditioned social distancing and isolation, which resulted in less frequent contacts with parents as compared to those under normal conditions. Therefore, from the academic point of view, it is recommended to perform new analyses in future studies. The results of such analyses would contribute to the development of the level of knowledge of work addiction and also would help capture differences in the interaction between the variables during crisis situations and under normal conditions.

## 5. Conclusions

Although the work addiction of parents has a promising potential in explaining the development of work addiction in adult children, it is insufficient for revealing the whole picture. Therefore, this study added the element of motivation to this explanation. The study confirmed most of our expectations and, importantly, indicated that perceived work addiction of the father was related to increased work addiction of an adult child through higher levels of extrinsic (rather than intrinsic) work motivation as a partial mediator.

## Figures and Tables

**Table 1:** Characteristics of the sample.

	Categories	Frequency	Percentage (%)
Gender	Male	161	30.0
	Female	376	70.0
Age	18–28 years	349	65.0
	29–61 years	188	35.0
Organizational tenure	Up to 4 years	418	77.8
	More than 4 years	119	22.2
Education	With higher education	429	79.9
	Without higher education	108	20.1
Occupational sector	Private	363	67.6
	Public	174	32.4

Note: age and organizational tenure groups are defined based on average scores (average age = 28.05; standard deviation (SD) = 6.84, average organizational tenure = 3.66; SD = 4.47).

**Table 2:** Reliability and validity of the instruments.

	HTMT Ratio							
	1	2	3	4	Standardized Factor Loadings	AVE	CR	Cronbach’s Alpha
1. Work addiction of adult child					0.42–0.78	0.52	0.88	0.84
2. Perceived work addiction of father	0.29				0.71–0.87	0.65	0.93	0.93
3. Perceived work addiction of mother	0.31	0.42			0.76–0.84	0.63	0.92	0.92
4. Intrinsic work motivation	0.29	0.05	0.10		0.90–0.96	0.86	0.95	0.92
5. Extrinsic work motivation	0.41	0.14	0.13	0.80	0.41–0.82	0.50	0.91	0.91

**Table 3:** Correlations, means and standard deviations of the study variables.

Variable	Mean	SD	1	2	3	4
1. Work addiction of adult child	2.81	0.83				
2. Work addiction of father	2.76	1.01	0.25 ***			
3. Work addiction of mother	2.81	0.98	0.27 ***	0.38 ***		
4. Intrinsic work motivation	5.38	1.50	0.20 ***	0.03	0.09 *	
5. Extrinsic work motivation	4.25	1.27	0.32 ***	0.14 **	0.13 **	0.72 ***

* *p* < 0.05, ** *p* < 0.01, *** *p* < 0.001.

## Data Availability

The data analyzed in this study may be obtained from the corresponding author upon reasonable request.

## References

[1] Gomes P., Diogo A., Santos E., Ratten V. (2022). Modeling the influence of workaholism on career success: A PLS–SEM approach. J. Manag. Organ..

[2] Soroka E., Iwanicka A., Olajossy M. (2020). Workaholism—Psychological and social determinants of work addiction. Curr. Probl. Psychiatry.

[3] Borges E.M.D.N., Sequeira C.A.D.C., Queirós C.M.L., Mosteiro-Díaz M.P. (2021). Workaholism and family interaction among nurses. Cien. Saude Colet..

[4] Ravoux H., Pereira B., Brousse G., Dewavrin S., Cornet T., Mermillod M., Mondillon L., Vallet V., Moustafa F., Dutheil F. (2018). Work addiction test questionnaire to assess workaholism: Validation of French version. JMIR Ment. Health.

[5] Ruiz-Garcia P., Castanheira A.M., Borges E., Mosteiro-Diaz M.P. (2022). Workaholism and work-family interaction among emergency and critical care nurses. Intensive Crit. Care Nurs..

[6] Hu Q., Schaufeli W., Taris T., Hessen D., Hakanen J.J., Salanova M., Shimazu A. (2014). East is east and west is west and never the twain shall meet: Work engagement and workaholism across eastern and western cultures. J. Behav. Soc. Sci..

[7] Balducci C., Menghini L., Conway P.M., Burr H., Zaniboni S. (2022). Workaholism and the enactment of bullying behavior at work: A prospective analysis. Int. J. Environ. Res. Public Health.

[8] Clark M.A., Michel J.S., Zhdanova L., Pui S.Y., Baltes B.B. (2016). All work and no play? A meta-analytic examination of the correlates and outcomes of workaholism. J. Manag..

[9] Kubota K., Shimazu A., Kawakami N., Takahashi M., Nakata A., Schaufeli W.B. (2010). Association between workaholism and sleep problems among hospital nurses. Ind. Health.

[10] Balducci C., Spagnoli P., Toderi S., Clark M.A. (2021). A within-individual investigation on the relationship between day level workaholism and systolic blood pressure. Work Stress.

[11] Clark M.A., Michel J.S., Stevens G.W., Howell J.W., Scruggs R.S. (2014). Workaholism, work engagement and work—Home outcomes: Exploring the mediating role of positive and negative emotions. Stress Health.

[12] Matsudaira K., Shimazu A., Fujii T., Kubota K., Sawada T., Kikuchi N., Takahashi M. (2013). Workaholism as a risk factor for depressive mood, disabling back pain, and sickness absence. PLoS ONE.

[13] Balducci C., Cecchin M., Fraccaroli F., Schaufeli W.B. (2012). Exploring the relationship between workaholism and workplace aggressive behaviour: The role of job-related emotion. Pers. Individ. Differ..

[14] Choi Y. (2013). The differences between work engagement and workaholism, and organizational outcomes: An integrative model. Soc. Behav. Pers..

[15] Gillet N., Morin A.J., Sandrin E., Houle S.A. (2018). Investigating the combined effects of workaholism and work engagement: A substantive-methodological synergy of variable-centered and person-centered methodologies. J. Vocat. Behav..

[16] Sandrin É., Gillet N., Fernet C., Depint-Rouault C., Leloup M., Portenard D. (2019). Effects of workaholism on volunteer firefighters’ performance: A moderated mediation model including supervisor recognition and emotional exhaustion. Anxiety Stress Coping.

[17] Kun B., Takacs Z.K., Richman M.J., Griffiths M.D., Demetrovics Z. (2021). Work addiction and personality: A meta-analytic study. J. Behav. Addict..

[18] An Y., Sun X., Wang K., Shi H., Liu Z., Zhu Y., Luo F. (2020). Core self-evaluations associated with workaholism: The mediating role of perceived job demands. Pers. Rev..

[19] Afota M.C., Robert V., Vandenberghe C. (2021). The interactive effect of leader-member exchange and psychological climate for overwork on subordinate workaholism and job strain. Eur. J. Work. Organ. Psychol..

[20] Mazzetti G., Schaufeli W.B., Guglielmi D. (2014). Are workaholics born or made? Relations of workaholism with person characteristics and overwork climate. Int. J. Stress Manag..

[21] Morkevičiūtė M., Endriulaitienė A., Poškus M.S. (2021). Understanding the etiology of workaholism: The results of the systematic review and meta-analysis. J. Workplace Behav. Health.

[22] Spagnoli P., Molino M., Molinaro D., Giancaspro M.L., Manuti A., Ghislieri C. (2020). Workaholism and technostress during the COVID-19 emergency: The crucial role of the leaders on remote working. Front. Psychol..

[23] Robinson B.E. (2014). Chained to the Desk.

[24] Chamberlin C.M., Zhang N. (2009). Workaholism, health, and self-acceptance. J. Couns. Dev..

[25] Kravina L., Falco A., Carlo N.A.D., Andreassen C.S., Pallesen S. (2014). Workaholism and work engagement in the family: The relationship between parents and children as a risk factor. Eur. J. Work Organ..

[26] Robinson B.E. (2000). Workaholism: Bridging the gap between workplace, sociocultural, and family research. J. Employ. Couns..

[27] Gagné M., Deci E.L. (2005). Self-determination theory and work motivation. J. Organ. Behav..

[28] Oates W.E. (1968). On being a workaholic. Pastor. Psychol..

[29] Clark M.A., Smith R.W., Haynes N.J. (2020). The multidimensional workaholism scale: Linking the conceptualization and measurement of workaholism. J. Appl. Psychol..

[30] Griffiths M.D., Demetrovics Z., Atroszko P.A. (2018). Ten myths about work addiction. J. Behav. Addict..

[31] Morkevičiūtė M., Endriulaitienė A. (2022). Defining the border between workaholism and work addiction: A systematic review. Int. J. Ment. Health Addict..

[32] Andreassen C.S., Griffiths M.D., Hetland J., Pallesen S. (2012). Development of a work addiction scale. Scand. J. Psychol..

[33] Griffiths M.D. (2005). A ‘components’ model of addiction within a biopsychosocial framework. J. Subst. Use.

[34] Andreassen C.S., Griffiths M.D., Sinha R., Hetland J., Pallesen S. (2016). The relationships between workaholism and symptoms of psychiatric disorders: A large-scale cross-sectional study. PLoS ONE.

[35] Kim S. (2019). Workaholism, motivation, and addiction in the workplace: A critical review and implications for HRD. Hum. Resour. Dev. Rev..

[36] Tremblay M.A., Blanchard C.M., Taylor S., Pelletier L.G., Villeneuve M. (2009). Work extrinsic and intrinsic motivation scale: Its value for organizational psychology research. Can. J. Behav. Sci..

[37] Stoeber J., Davis C.R., Townley J. (2013). Perfectionism and workaholism in employees: The role of work motivation. Pers. Individ. Differ..

[38] Broeck A.V.D., Schreurs B., Witte H.D., Vansteenkiste M., Germeys F., Schaufeli W. (2011). Understanding workaholics’ motivations: A self-determination perspective. J. Appl. Psychol..

[39] Taris T.W., van Beek I., Schaufeli W.B. (2020). The motivational make-up of workaholism and work engagement: A longitudinal study on need satisfaction, motivation, and heavy work investment. Front. Psychol..

[40] Beek I.V., Hu Q., Schaufeli W.B., Taris T.W., Schreurs B.H. (2012). For fun, love, or money: What drives workaholic, engaged, and burned-out employees at work?. J. Appl. Psychol..

[41] Wojdylo K., Baumann N., Kuhl J. (2017). The firepower of work craving: When self-control is burning under the rubble of self-regulation. PLoS ONE.

[42] Andreassen C.S. (2014). Workaholism: An overview and current status of the research. J. Behav. Addict..

[43] Endriulaitienė A., Morkevičiūtė M. (2020). The unintended effect of perceived transformational leadership style on workaholism: The mediating role of work motivation. J. Psychol..

[44] Kang S. (2020). Workaholism in Korea: Prevalence and socio-demographic differences. Front. Psychol..

[45] Fritz M.S., MacKinnon D.P. (2007). Required sample size to detect the mediated effect. Psychol. Sci..

[46] Hu L.T., Bentler P.M. (1999). Cutoff criteria for fit indexes in covariance structure analysis: Conventional criteria versus new alternatives. Struct. Equ. Model..

[47] Wasserstein R.L., Schirm A.L., Lazar N.A. (2019). Moving to a world beyond “*p* < 0.05”. Am. Stat..

[48] Hair J.F., Black W.C., Babin B.J., Anderson R.E., Tatham R.L. (2010). Multivariate Data Analysis.

[49] Nunnally J.C., Bernstein I. (1994). Elements of Statistical Description and Estimation, Psychometric Theory.

[50] Henseler J., Ringle C.M., Sarstedt M. (2015). A new criterion for assessing discriminant validity in variance-based structural equation modeling. J. Acad. Mark. Sci..

[51] Podsakoff P.M., MacKenzie S.B., Lee J.Y., Podsakoff N.P. (2003). Common method biases in behavioral research: A critical review of the literature and recommended remedies. J. Appl. Psychol..

[52] Burnham K.P., Anderson D.R. (2004). Multimodel inference: Understanding AIC and BIC in model selection. Sociol. Methods Res..

[53] Tóth-Király I., Morin A.J., Salmela-Aro K. (2021). A longitudinal perspective on the associations between work engagement and workaholism. Work Stress.

[54] American Psychiatric Association (2018). Diagnostic and Statistical Manual of Mental Disorders.

[55] Spector P.E. (2019). Do not cross me: Optimizing the use of cross-sectional designs. J. Bus. Res..

[56] Mónico L.S., Margaça C. (2021). The workaholism phenomenon in Portugal: Dimensions and relations with workplace spirituality. Religions.

